# Social processing modulates the initial allocation of attention towards angry faces: evidence from the N2pc component

**DOI:** 10.1093/scan/nsad070

**Published:** 2023-11-16

**Authors:** Benedikt Emanuel Wirth, Dirk Wentura

**Affiliations:** Department of Psychology, Saarland University, Campus A2 4, Saarbrücken 66123, Germany; Cognitive Assistants Department, German Research Center for Artificial Intelligence (DFKI), Campus D3 1, Saarbrücken 66123, Germany; Department of Psychology, Saarland University, Campus A2 4, Saarbrücken 66123, Germany

**Keywords:** angry faces, attentional bias, dot-probe task, N2pc component, social processing

## Abstract

Previous research has shown that attentional bias towards angry faces is moderated by the activation of a social processing mode. More specifically, reliable cueing effects for angry face cues in the dot-probe task only occurred when participants performed a task that required social processing of the target stimuli. However, cueing effects are a rather distal measure of covert shifts in spatial attention. Thus, it remains unclear whether the social processing mode modulates initial allocation of attention to or attentional disengagement from angry faces. In the present study, we used the N2pc, an event-related potential component, as an index of attentional shifts towards angry faces. Participants performed a dot-probe task with two different target conditions while the electroencephalogram (EEG) was recorded. In the social target condition, target stimuli were socially meaningful (schematic faces), and in the non-social target condition, they were meaningless (scrambled schematic faces). The amplitude of the N2pc component elicited by angry face cues was significantly larger in the social target condition than in the non-social target condition. This pattern also occurred for behavioural cueing effects. These results suggest that the activation of a social processing mode due to current task demands affects the initial allocation of attention towards angry faces.

Facial expressions of emotion are important social signals in everyday life, which can convey important information on otherwise ambiguous situations to the observer. For example, a smile can convey security or affiliation, a frown scepticism or outright rejection, and an angry snarl can signal immediate danger to the observer. Therefore, many theories of emotional attention argue that human visual attention is biased towards facial expressions of emotion—especially towards threatening ones, such as fear and anger. Some theories argue that this bias is caused by the importance of emotional expressions during human phylogeny (Öhman and Mineka, 2001). Others argue that facial expressions capture attention due to their highly arousing nature (Anderson, 2005). A third group claims that the general relevance to the observer is the critical factor causing this bias (Brosch *et al.*, 2008). Despite their differences, all the aforementioned theories assume that an attentional bias towards emotional expressions is an adaptive process found in all humans. In contrast, clinical models of anxiety argue that attentional bias towards emotional expressions (and other types of threatening stimuli) is a maladaptive process only found in highly anxious individuals (Bar-Haim *et al.*, 2007). Some clinical theories even argue that the bias might be a causal factor in the aetiology of anxiety disorders (van Bockstaele *et al.*, 2014).

A growing number of studies propose an intermediate view on this issue: these studies argue that the general population (i.e. predominantly non-anxious individuals) can show an attentional bias towards emotional stimuli (like facial expressions), but that this bias is not unconditional (i.e. not caused purely by bottom-up characteristics of these stimuli). Rather, these studies suggest that attentional bias towards emotional faces is contingent on top-down processes of the observer, which are activated by specific task demands (Barratt and Bundesen, 2012; Everaert *et al.*, 2013; Vromen *et al.*, 2015, 2016; Glickman and Lamy, 2018; Puls and Rothermund, 2018; Wirth and Wentura, 2018a, 2019; Victeur *et al.*, 2019; Vogt *et al.*, 2022). The specific nature of these top-down processes is, however, currently unclear. Some studies argue that an attentional bias towards emotional stimuli only occurs if the current task requires participants to search for or respond to a stimulus of exactly the same category. For example, Victeur *et al.* (2019) found that participants only show a bias towards fearful faces if they have to respond to fearful faces (see Vromen *et al.*, 2015, 2016, for two studies making a similar argument for threatening spider stimuli). Another study argues that emotional stimuli capture visual attention if participants are in an affective processing mode because the classification of affective information is required in the current task (Everaert *et al.*, 2013). A further study argues that emotional faces only capture attention via an indirect top-down process: when participants are searching for a target that is defined by a unique feature (i.e. a singleton target), emotional faces with a unique emotional expression among a crowd of neutral faces (i.e. emotional singletons) capture visual attention (Glickman and Lamy, 2018).

Specifically, for the stimulus class of angry faces, we recently identified another candidate process that could explain the occurrence (or absence) of an attentional bias, namely, social processing (Wirth and Wentura, 2018a, 2019). More precisely, we argue that non-anxious individuals only show an attentional bias towards angry faces if the current task (or in more general terms: the current situation/context) requires the observer to process social information from the environment. We corroborated this claim in two studies (Wirth and Wentura, 2018a, 2019) using different variants of the dot-probe task.

For example, in Experiment 1 of Wirth and Wentura (2019), each trial consisted of a cue display and a target display (see also Figure 1 for an illustrative depiction). During the cue display, two photographic face cues were presented left and right of fixation, one angry and one neutral. One-hundred milliseconds after the onset of the cue display, the target display was presented. It also contained two stimuli that were presented left and right of fixation, one target stimulus and one distractor stimulus. Participants’ task was to categorise the target as quickly as possible while ignoring the distractor. Crucially, the experiment consisted of two blocks: in the social target block, the target and distractor stimuli were socially meaningful (schematic faces) and participants had to find the target based on a socially relevant dimension (open mouth *vs* closed mouth). In contrast, in the non-social target block, the target and distractor stimuli were not socially meaningful (scrambled schematic faces), and the target had to be identified based on a non-social dimension (horizontal double line *vs* single horizontal line). The angry face cue only produced significant cueing effects (i.e. faster responses when the target was presented in the same location as the angry face cue than when it was presented in the opposite location) during the social target block, but not during the non-social target block. We interpreted this finding in the following terms: because participants were required to actively process social information in the social target block, a social processing mode was activated. This social processing mode, in turn, caused an attentional bias towards angry faces. In contrast, during the non-social target block, no such processing mode was activated, and consequently, no bias towards angry faces occurred.

**Fig. 1.**
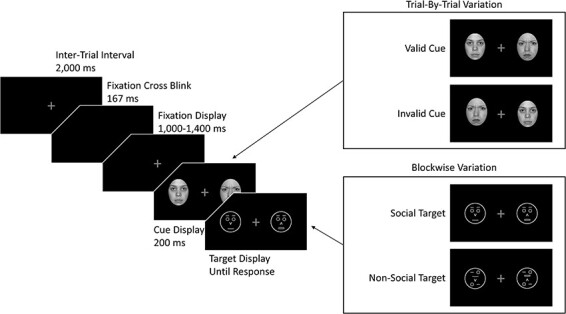
Illustration of a typical trial and the design of the experiment. For the sake of visibility, proportions are not true to scale.

However, a prevalent criticism of the dot-probe task in general is that it relies on a rather distal measure of the attentional bias, namely, target response times (RTs). The use of this distal measure leaves it unclear, which specific attentional processes are reflected by any cueing effects that might be found with the dot-probe task (Fox *et al.*, 2001; Koster *et al.*, 2004; Salemink *et al.*, 2007; Cisler *et al.*, 2009; Cisler and Koster, 2010; Clarke *et al.*, 2013; Grafton and MacLeod, 2014; Rudaizky *et al.*, 2014; Müller *et al.*, 2016). On the one hand, it could be the case that these cueing effects reflect a bias in the initial allocation of attention: participants are more likely to initially allocate their attention to the emotional stimulus (i.e. the angry face in our previous study) than to the neutral stimulus (i.e. the neutral face in our previous study). Thus, when the target is presented in the same location as the emotional stimulus (valid trials), participants’ attention is already in the optimal location to process the target once it appears. In contrast, when the target is presented in the opposite location of the emotional stimulus (invalid trials), participants’ attention needs to be shifted to the other location to process the target—a process that takes additional time.

On the other hand, however, cueing effects measured in the dot-probe task might reflect difficulties in disengaging attention from emotional stimuli. Thus, the probability of initial attention allocation might be the same for emotional and neutral stimuli. However, when attention is allocated to the neutral cue stimulus, participants can reorient their attention back to the central fixation cross before the onset of the target display. In contrast, when attention is randomly allocated to the emotional stimulus first, participants have problems in disengaging attention from the stimulus (i.e. the stimulus ‘holds’ attention). Thus, participants’ attention is still in the location of the emotional stimulus when the target display is presented. Consequently, participants’ attention will be in the optimal location to process the target when it is presented in the same location as the emotional stimulus (valid trials), but not when it is presented in the opposite location (invalid trials).

Thus, the finding that the activation of a social processing mode modulates attentional bias towards angry faces (Wirth and Wentura, 2018a, 2019) could potentially reflect one of the following two processes: first, only when a social processing mode is activated (due to current task demands), participants’ attention is preferentially allocated to angry (as opposed to neutral) face cues. Second, even when a social processing mode is activated, participants’ attention is equally likely to be initially allocated to the neutral or angry face cue. However, the social processing mode causes a delayed disengagement of attention from angry face cues. In the present study, we aimed to disentangle these two processes using the N2pc, an event-related potential (ERP) component, as a more proximal measure of spatial shifts in visual attention.

The N2pc component reflects the focusing of covert attention on a peripheral stimulus and the filtering of other stimuli (see Luck, 2012 for a review). It is manifested by an increased negativity at posterior electrodes contralateral to the attended stimulus compared to posterior electrodes ipsilateral to the attended stimulus ∼180–300 ms after the stimulus onset. The N2pc is mainly used to investigate attention towards potential target items during visual search—that is, stimuli that gain attentional priority due to top-down processes. However, it has also been shown that stimuli that gain attentional priority due to bottom-up processes like highly salient colour stimuli (colour popouts) elicit a reliable—albeit substantially smaller—N2pc (e.g. Luck and Hillyard, 1994; Hickey *et al.*, 2006). Accordingly, the N2pc has been shown to be reliably elicited by task-irrelevant emotional stimuli such as threatening pictures from the International Affective Picture System (Kappenman *et al.*, 2014, 2015), fearful faces (Eimer and Kiss, 2007; Fenker *et al.*, 2010) and angry faces (Holmes *et al.*, 2009; Reutter *et al.*, 2017).

Therefore, the present study aims to use the N2pc component in order to clarify whether the social processing mode affects initial allocation of attention towards angry faces or whether it causes delayed disengagement from angry faces. If the social processing mode affects the initial allocation of attention, then participants should show an enhanced N2pc for angry face cues when performing a task that requires social processing. In contrast, if the social processing mode affects attentional disengagement, then the N2pc component for angry face cues should be similar during a task that requires social processing and a task that does not. In both cases, we expect to find a modulation of participants’ RT-based cueing effects (i.e. larger cueing effects in the social task than in the non-social task) in accordance with previous studies (Wirth and Wentura, 2018a, 2019).

It should be added that even a significant modulation of the N2pc by the social character of the task cannot rule out the possibility that the social processing mode additionally affects attentional disengagement. Therefore, we additionally analysed the post-N2pc positivity (PNP), an ERP component that has recently been discussed as a marker of attentional disengagement (Sawaki *et al.*, 2012; Papaioannou and Luck, 2020; Drisdelle and Eimer, 2021). The PNP—also referred to as distractor positivity (*P*_D_)—is manifested by a contralateral positivity that follows the N2pc.

## Behavioural pilot study

In previous experiments (Wirth and Wentura, 2018a, 2019), we used a cue-target onset asynchrony (CTOA) of 100 ms. However, with such a short CTOA, the N2pc component elicited by the cue display temporally overlaps with the P1 component elicited by the target display. Thus, any modulations of the cue-locked N2pc could be contaminated by modulations of the target-locked P1.^1^ Therefore, we decided to increase the CTOA to 200 ms. CTOAs of 200 ms (Grubert and Eimer, 2016) and 250 ms (Goller *et al.*, 2020) have successfully been used in the past to study the N2pc. However, increasing the CTOA makes the detection of RT-based cueing effects potentially less likely. Since the complete absence of RT-based cueing effects would make any interpretation of the results very difficult (even if significant effects for the N2pc occurred), we conducted a behavioural pilot study where *N* = 75 participants performed a dot-probe task similar to Experiment 1 in Wirth and Wentura (2019) but with a CTOA of 200 ms. We calculated cueing scores by subtracting the average RT on valid trials from the average RT on invalid trials for each participant. Thus, a positive cueing score indicates an attentional bias towards angry face cues. Consistent with our previous results, we found significantly positive cueing scores for angry face cues in the social target condition, *M* = 7 ms, *t*(74) = 3.15, *P* = 0.002, *d*_Z_ = 0.36, but not in the non-social target condition, *M* = 2 ms, *t*(74) = 0.68, *P* = 0.496, *d*_Z_ = 0.08. Interested readers are referred to the Supplementary Materials for a detailed description of the methods and results of the pilot study.

## ERP study

### Method

#### Participants

Fifty-three university students received €20.00 for their participation. Three participants were excluded from all further analyses because their overall accuracy in at least one of the experimental blocks was more than three interquartile ranges below the first quartile of the distribution of all participants (Tukey, 1977). Of the remaining *N* = 50 participants, 10 were male and 40 were female. Age ranged from 18 to 31 years (*M *= 24.6, *SD* = 3.1). All participants reported normal or corrected-to-normal vision and provided informed consent prior to testing. Two additional participants had to be excluded from the EEG analyses—in one case, because the reference electrode went defunct during recording, which caused excessive noise, and in the other case, because the EEG data were accidentally not recorded throughout the largest part of the experiment. Thus, a sample size of *N* = 48 remained for the ERP analyses.

The sample size was determined according to the following considerations: we expected an effect size of *d*_Z_ = 0.40 for the moderation of the N2pc component to angry faces by the social character of the targets. This effect size is a bit larger than the effect size of approximately *d*_Z_ = 0.30 that we found for behavioural data in previous studies (Wirth and Wentura, 2018a, 2019). However, we increased the number of trials from 448 in previous studies to 896 in the present study to increase the signal-to-noise ratio, which should also lead to increased effect sizes. According to G*Power (Faul *et al.*, 2007), given an α = 0.05, 52 participants are needed to detect an effect of this size with a power of 1 − ß = 0.80 (power slightly decreased after outlier exclusion to 1 − ß = 0.79 with *N* = 50).

#### Design

The design was a 2 (target type: social target *vs* non-social target) × 2 (cue validity: valid angry cue *vs* invalid angry cue) design with target type as a block-wise within-subjects factor and cue validity as a trial-by-trial within-subjects factor. Furthermore, we measured participants’ trait anxiety as a covariate. As in previous studies (Wirth and Wentura, 2018a, 2019), we did not expect any moderation of participants’ attentional bias towards angry face cues by trait anxiety. However, since our aim was to investigate the moderation of attentional bias towards angry faces in the general (i.e. non-anxious) population, we had to ascertain that any potential effects are not solely driven by the more anxious participants in our sample.

#### Materials

As cue stimuli, we selected the same photographs of eight female and eight male individuals showing angry and neutral expressions as in our previous experiments (Wirth and Wentura, 2018a, 2019). We only included faces with closed mouths from the NimStim set of facial expressions (Tottenham *et al.*, 2009) because (i) ERPs are highly sensitive to perceptual low-level characteristics and could be affected by the high luminance and contrast of exposed teeth and (ii) we showed that exposed teeth can potentially distort RT-based cueing effects in the dot-probe task (Wirth and Wentura, 2018b). Using Adobe Photoshop (Adobe Systems Inc., San Jose, CA), all stimuli were cropped into a standard oval shape concealing hair and external features and were converted to greyscale (Figure 1). Participants’ trait anxiety was measured with a computerised version of the trait scale of the German version of the State-Trait Anxiety Inventory (STAI; Laux *et al.*, 1981). This self-assessment scale contains 20 items, each scored between 1 (low anxiety) and 4 (high anxiety).

#### Procedure

The study was conducted on a computer equipped with a 23″ EIZO FS2331 monitor (Eizo Corporation, Hakusan, Japan) using a resolution of 1920 × 1080 pixels, a refresh rate of 60 Hz and a colour depth of 32 bit. The experimental routine was programmed using Psychtoolbox-3 (Kleiner *et al.,* 2007) for Matlab (Mathworks, Natick, MA).

Participants were seated ∼65 cm from the monitor and were presented with an instruction screen explaining the experimental procedure. Figure 1 depicts a schematic illustration of a typical trial and the design of the experiment. Throughout the procedure, a grey fixation cross was presented on a black background to maintain participants’ focus on the centre of the screen. To indicate the beginning of a trial, the fixation cross blinked for 167 ms. The fixation cross then remained on screen for a variable interval spanning from 1000 to 1400 ms (in steps of 100 ms) to avoid any anticipatory effects. Subsequently, two photographic face cues, one angry and one neutral, were presented laterally for 200 ms. Each face had a size of 4.5 × 6.2 cm (4.0 × 5.5°); the centre-to-centre distance between the faces was 11.1 cm (9.8°). Immediately after the offset of the cues, two white line drawings—one target stimulus and one distractor stimulus—appeared at the cue positions and remained there until a response was given.

In the social target condition, these stimuli were schematic faces with a neutral expression—the target face had an open mouth (indicated by a double line) and the distractor face had a closed mouth (indicated by a single line). Participants’ task was to indicate the direction in which the nose of the schematic target face pointed (up or down) while ignoring the distractor face. In the non-social target condition, scrambled versions of the schematic faces were presented. These scrambled faces consisted of the same basic features as the schematic faces, but the spatial configuration of those features was altered (i.e. the mouth was located above the nose, one eye and one eyebrow were located below the nose; Figure 1). Thus, the scrambled schematic faces conveyed the impression of a complex, meaningless pattern surrounded by a circle. Participants’ task was to find the (target) pattern that contained a horizontal double line (corresponding to the open mouth in the social target condition) and indicate whether the arrow in this pattern (corresponding to the nose in the social target condition) was pointing up or down. Moreover, participants were told to ignore the arrow in the (distractor) pattern, which contained only a single horizontal line (corresponding to the closed mouth of the distractor face in the social target condition).

The schematic faces/scrambled faces had a size of 2.8 × 2.8 cm (2.5 × 2.5°) and the centre-to-centre distance between them was 11.1 cm (9.8°). Nose/arrow directions of the target and distractor stimuli were uncorrelated, that is, the nose/arrow of the target stimulus pointed in the same direction as the nose/arrow of the distractor stimulus in 50% of the trials and in the opposite direction in the remaining trials (this was varied orthogonally to the other experimental factors). Participants were asked to respond as fast as possible by pressing the ‘t’ key for ‘up’ or the ‘v’ key for ‘down’ on a standard German QWERTZ keyboard. In half of the trials, the target stimulus appeared at the location of the angry face cue (valid cue), and in half of the trials, it appeared at the location of the neutral face cue (invalid cue). Each response was followed by a 2000-ms inter-trial interval. If participants made an error or took longer than 1500 ms to submit a response, the computer produced a 1000 Hz warning tone of 500 ms duration.

The experiment comprised 896 trials that were presented in two blocks consisting of 448 trials each—one with schematic faces as the target and distractor stimuli and the other with scrambled faces as the target and distractor stimuli. Twenty-five participants completed the social target block first and the remaining 25 participants completed the non-social target block first. Within each block, a self-paced break was included every 56 trials. At the beginning of each block, participants were presented with 32 training trials that contained faces not presented during the main trials. These training trials were not included in the data analysis. At the end of the experiment, participants completed the trait anxiety scale of the STAI (Laux *et al.*, 1981). The whole procedure (including preparation of the participants) lasted ∼150 minutes.

#### EEG recording and pre-processing

EEG was recorded with a BrainAmps Direct Current amplifier (upper cut-off frequency 250 Hz, sampling rate 1000 Hz) and active Ag–AgCl electrodes mounted on an elastic cap from 29 scalp sites according to the extended international 10–20 system: Fpz, F7, F3, Fz, F4, F8, FC5, FC1, FC2, FC6, C3, Cz, C4, TP9, CP5, CP1, CP2, CP6, TP10, P7, P3, Pz, P4, P8, PO7, PO8, Oz, PO9 and PO10. Horizontal electrooculograms were recorded bipolarly from the outer canthi of both eyes. An electrode placed on the left earlobe served as the reference for online recording, and EEG was re-referenced offline to the average of both earlobes.

Pre-processing was conducted using EEGLAB (Delorme and Makeig, 2004) and ERPLAB (Lopez-Calderon and Luck, 2014). First, the continuous EEG signal was high-pass filtered at 0.5 Hz, and the (European) line frequency and its harmonics (50 Hz, 100 Hz, 150 Hz) were removed using the CleanLine plugin (https://www.nitrc.org/projects/cleanline). EEG was epoched offline from 100 ms before to 500 ms after the onset of the cue display on each trial. For each EEG epoch, amplitude values were computed relative to a 100 ms baseline prior to cue onset. Epochs from trials with incorrect responses or RTs that were more than 1.5 interquartile ranges above the third quartile of the individual participant’s RT distribution in the respective block were excluded from all further analyses (Tukey, 1977)—both behavioural and EEG analyses. Across all participants, 3.0% of trials with correct responses were excluded due to slow RTs. Furthermore, epochs with non-ocular artefacts were manually rejected. This led to the exclusion of 3.8% of all epochs across participants. Subsequently, ocular artefacts were corrected using independent component analysis. EEG waveforms were averaged separately for all combinations of target type (social target *vs* non-social target) and visual field of the angry face cue (left *vs* right). N2pc components were quantified on the basis of mean amplitudes obtained in the time window ranging from 180 to 300 ms after the cue onset at lateral posterior electrodes PO9 and PO10. The time window was selected a priori based on previous literature (Mazza *et al.*, 2013; Reutter *et al.*, 2017; Stoletniy *et al.*, 2023). PNP amplitudes were quantified on the basis of mean amplitudes obtained in the time window ranging from 380 to 500 ms after the cue onset at lateral posterior electrodes PO9 and PO10. In contrast to the time window of the N2pc, the time window of the PNP was selected a posteriori after visual inspection of the contra-ipsi waveforms.

### Results

#### Behavioural results

Across all experimental conditions, average classification accuracy was *M* = 95.1% (*SD* = 4.4). Individual RTs (after outlier removal) ranged from *M* = 541 to *M* = 912 (the grand mean was *M* = 760, *SD* = 84).

A 2 (target type) × 2 (cue validity) within-subjects analysis of variance (ANOVA) with RTs as the dependent variable yielded a significant main effect of cue validity, *F*(1, 49) = 15.97, *P* < 0.001, η_p_^2^ = 0.246, with RTs being shorter on valid trials (*M* = 756 ms, *SD* = 85) than on invalid trials (*M* = 763 ms, *SD* = 83). The main effect of target type did not reach significance, *F*(1, 49) = 0.28, *P* = 0.599, η_p_^2^ = 0.006. Importantly, however, a significant target type × cue validity interaction occurred, *F*(1, 49) = 4.88, *P* = 0.032, η_p_^2^ = 0.091. As in previous studies (Wirth and Wentura, 2018a, 2019), additionally including participants’ trait anxiety as a (centred) covariate did not yield any significant effects involving the covariate, all *F*(1, 48) < 3.31, all *P* > 0.075, all η_p_^2^ < 0.065.

**Fig. 2.**
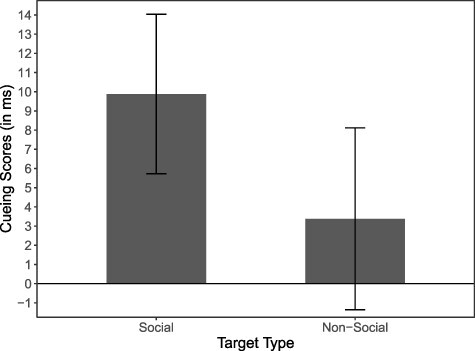
Average cueing scores for social target and non-social target trials. Cueing scores represent the difference between the average reaction times to invalidly cued trials and validly cued trials (error bars depict the 95% confidence interval).

To further scrutinise the interaction between target type and cue validity, we calculated cueing scores by subtracting the average RT on valid trials from the average RT on invalid trials for each participant. As can be seen in Figure 2, cueing scores were substantially larger in the social target condition (*M* = 10 ms, *SE* = 2) than in the non-social target condition (*M* = 3 ms, *SE* = 2). Cueing scores in the social target condition significantly differed from zero, *t*(49) = 4.78, *P* < 0.001, *d*_Z_ = 0.67, while cueing scores in the non-social target condition did not, *t*(49) = 1.43, *P* = 0.159, *d*_Z_ = 0.20.

#### ERP results

**Fig. 3.**
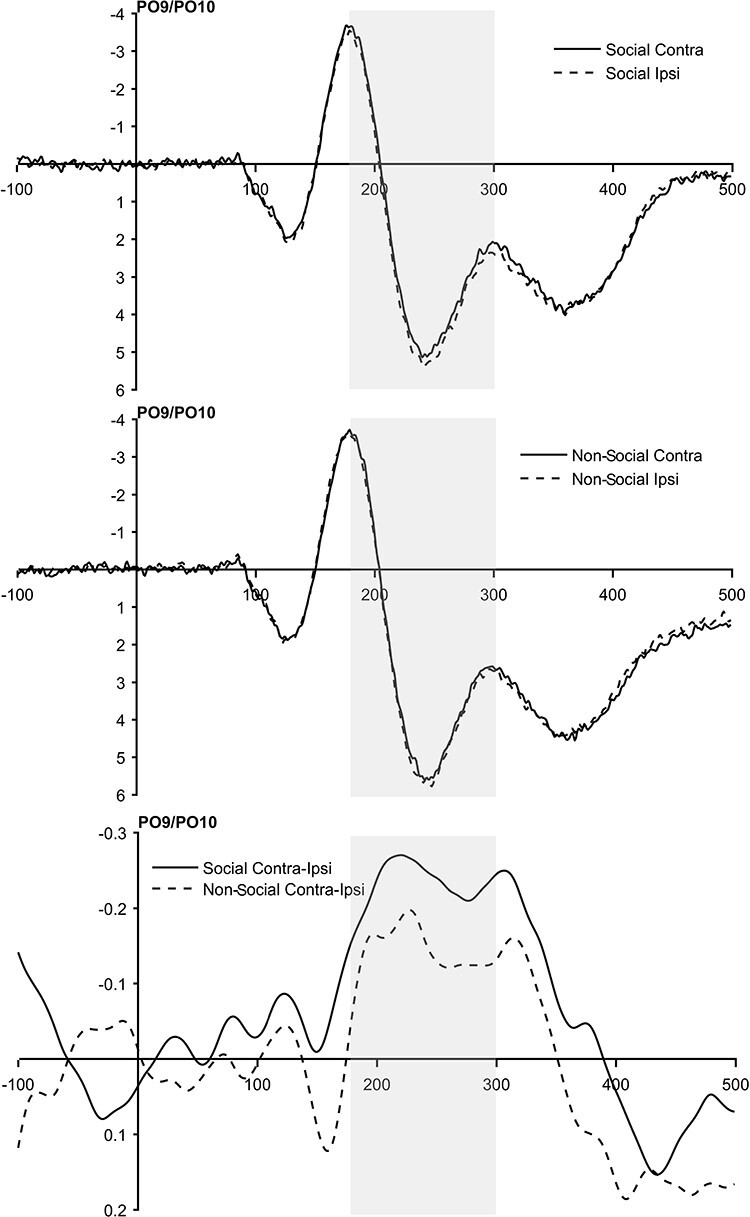
Grand-average ERPs measured at electrode sites PO9 and PO10 contralateral and ipsilateral to the location of the angry face cue, separately for the social target condition (top panel) and the non-social target condition (mid panel). The difference between contralateral and ipsilateral waveforms (bottom panel) in the 180–300 ms interval after the cue onset is larger in the social target condition than in the non-social target condition. For the sake of visual clarity, difference waveforms were smoothed using a non-causal Butterworth low-pass filter (half-amplitude cut-off = 20 Hz, slope = 24 dB/octave).

Figure 3 shows grand-average ERPs elicited at electrode sites PO9 and PO10 contra- and ipsilateral to angry face cues, separately for social target and non-social target trials in the 500-ms interval after the onset of the cue display as well as the difference between contralateral and ipsilateral waveforms. In the 180–300 ms interval after cue onset, negativity was higher at electrode sites contralateral to the angry face cues than at electrode sites ipsilateral to the angry face cues. Interestingly, this difference was more pronounced in the social target condition than in the non-social target condition.

**Fig. 4.**
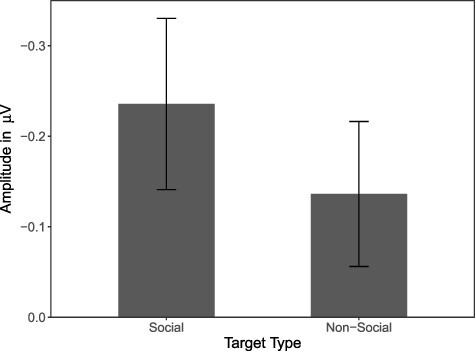
Average N2pc amplitudes for social target and non-social target trials. N2pc amplitudes reflect the difference in electric potential obtained by subtracting ERPs ipsilateral to the angry face cue from ERPs contralateral to the angry face cue (error bars depict the 95% confidence interval).

In order to statistically validate this result, we calculated a 2 × 2 within-subjects ANOVA with the factors target type (social target *vs* non-social target) and hemisphere (ipsilateral to angry cue *vs* contralateral to angry cue) and mean amplitude as the dependent variable. The ANOVA yielded a significant main effect of hemisphere, *F*(1, 47) = 25.28, *P* < 0.001, η_p_^2^ = 0.350, indicating that across target conditions, angry face cues elicited a significant N2pc. The main effect of target type did not reach significance, *F*(1, 47) = 2.75, *P* = 0.104, η_p_^2^ = 0.055. Importantly, however, a significant target type × hemisphere interaction occurred, *F*(1, 47) = 4.66, *P* = 0.036, η_p_^2^ = 0.090. In order to scrutinise this interaction, we calculated N2pc amplitudes by subtracting ipsilateral ERPs from contralateral ERPs. As can be seen in Figure 4, mean N2pc amplitudes were substantially larger in the social target condition than in the non-social target condition. Amplitudes both in the social target condition, *t*(47) = 5.01, *P* < 0.001, *d*_Z_ = 0.72, and in the non-social target condition, *t*(47) = 3.42, *P* = 0.001, *d*_Z_ = 0.49, differed significantly from zero. As in the behavioural analyses, additionally including participants’ trait anxiety as a (centred) covariate did not yield any significant effects involving the covariate, all *F*(1, 46) < 2.09, all *P* > 0.155, all η_p_^2^ < 0.044.^2^

Furthermore, we calculated a corresponding ANOVA to investigate the effect of social processing on mean PNP amplitudes. This analysis yielded significant main effects of task, *F*(1, 47) = 28.54, *P* < 0.001, η_p_^2^ = 0.378, and of hemisphere, *F*(1, 47) = 18.62, *P* < 0.001, η_p_^2^ = 0.284. Importantly, however, no significant target type × hemisphere interaction occurred, *F*(1, 47) = 2.59, *P* = 0.114, η_p_^2^ = 0.052. Thus, while N2pc components were followed by significant PNP components (as indicated by the significant main effect of hemisphere), the amplitudes of these components were not affected by target type.

## Discussion

In previous studies, we found that attentional bias towards angry faces is moderated by a social processing mode (Wirth and Wentura, 2018a, 2019). In the present study, we investigated whether this social processing mode affects the initial allocation of attention using the N2pc component as a measure of lateral shifts in spatial attention. Participants performed a dot-probe task with two target conditions. The social target condition used socially meaningful target stimuli (schematic faces), whereas the non-social target condition used meaningless target stimuli (scrambled schematic faces). As in our previous studies, participants showed significantly larger cueing effects in the social target condition than in the non-social target condition. Interestingly, the cueing effect for angry faces in the social target condition was substantially larger in the present study (*d*_Z_ = 0.67) than in our previous studies (*d*_Z_ = 0.34 across all previous experiments). One potential reason for this larger effect is a better signal-to-noise ratio due to the increased number of trials per target condition from 224 in previous studies to 448 in the present study.

Importantly, a pattern similar to the pattern found for cueing effects also occurred for the N2pc component: mean N2pc amplitudes were significantly larger in the social target condition than in the non-social target condition. This pattern suggests that the social processing mode affects the initial allocation of attention towards angry faces. In fact, the effect size of the interaction was almost identical for the cueing effects and for the N2pc amplitudes (both *d*_Z_ = 0.32). This interpretation is also consistent with the absence of any significant modulation of PNP amplitudes by the social character of the performed task. This finding suggests that the social processing mode does not affect attentional disengagement from angry faces.

It should further be noted that our results suggest that attentional bias towards angry faces is not an all-or-nothing phenomenon (which is present in the social target condition and completely absent in the non-social target condition). Rather, participants also showed a significant—albeit smaller—N2pc component to angry faces in the non-social target condition. The absence of any significant behavioural cueing effects in the present and previous (Wirth and Wentura, 2018a, 2019) studies might therefore not be a consequence of the complete absence of an attentional bias but rather a consequence of RT-based cueing effects being a rather distal—hence noisier—measure of the attentional bias. Only if the attentional bias towards angry faces reaches a certain strength, it becomes apparent in RT-based cueing effects.

This interpretation is consistent with an exploratory post hoc analysis: we ordered RTs of individual trials from fast to slow and grouped them into four discrete bins (separately for each participant and each combination of experimental variables). For each of the four bins, we then calculated cueing scores and used one-sided *t*-tests to assess whether the cueing scores were significantly different from zero. In the social target condition, cueing scores in all four bins significantly differed from zero. In contrast, in the non-social target condition, cueing scores were only significantly different from zero in the two fastest bins.

In basic attention research, there has been a long-standing debate on the question under which conditions specific stimuli involuntarily capture spatial attention (see Luck *et al.*, 2021 for a recent review). Stimulus-driven accounts argue that certain kinds of physically salient stimuli (e.g. abrupt onsets, colour singletons, etc.) automatically capture visual attention even when completely task irrelevant (Theeuwes, 1992, 2004; Belopolsky *et al.*, 2010). Empirical support for this claim comes mainly from the additional singleton paradigm: for example, when participants are searching for a red diamond among red circles, they are slower to find the red diamond when one of the circle distractors is green than when all circle distractors are red—even though colour is a completely task-irrelevant feature. Proponents of stimulus-driven accounts argue that this increase in search times caused by the presence of an additional, task-irrelevant singleton reflects initial capture of the highly salient colour singleton and subsequent redirection of attention to the target (see, however, Wirth *et al.*, 2023 for a recent critique of this interpretation). In contrast, contingent capture theory (Folk *et al.*, 1992; Folk and Remington, 1998, 2006) argues that even highly salient stimuli like colour singletons or abrupt onsets only capture attention when they match the target in a task-relevant feature—or to be more precise: when the feature relationship between the critical cue and the other items on the cue display matches the feature relationship between the target and the non-targets (Becker *et al.*, 2013). Support for contingent capture theory mainly comes from the exogenous spatial-cueing paradigm: for example, when participants are searching for green targets, attention is only captured by green cues, but not by red cues (and vice versa).

Both these extreme positions cannot account for our results very well: if attentional bias towards angry faces was completely stimulus driven (due to the threatening nature of angry faces), there should be no modulation of the bias by the current task. On the other hand, if attentional bias towards angry faces was completely goal contingent, no attentional bias (i.e. no significant N2pc components) should occur in the non-social target condition.^3^ However, our finding of a quantitative boost of attentional bias towards angry faces by the activation of a social processing mode is consistent with priority-based accounts of spatial attention (Lamy *et al.*, 2018; Wolfe, 2021; Darnell and Lamy, 2022) that assume that (i) both bottom-up (i.e. stimulus driven) and top-down (i.e. goal contingent) processes affect spatial attention and (ii) attentional capture is not an all-or-nothing process: angry faces gain attentional priority via bottom-up processes due to their threatening nature. However, based on these bottom-up processes alone, the competitive edge of angry faces over neutral faces is not sufficient to produce reliable cueing effects (but sufficient to produce reliable N2pc components). However, when a social processing mode is activated, it seems that angry faces additionally gain attentional priority via top-down processes. The combined bottom-up and top-down activation is then sufficient to produce reliable cueing effects.

It should be noted, however, that the attentional priority of angry faces (over neutral faces) is generally rather small. For example, the amplitude of the N2pc towards potential target items during visual search is usual in the range of 1–3 μV (e.g. Grubert and Eimer, 2013, 2016; Zivony *et al.*, 2018). In contrast, the amplitude of the N2pc elicited by angry face cues in the present study was only the fraction of a microvolt. However, this is within the range of amplitudes found for the N2pc elicited by emotional faces observed in previous studies (e.g. Eimer and Kiss, 2007; Holmes *et al.*, 2009; Reutter *et al.*, 2017).

In conclusion, the present study shows that the N2pc component elicited by angry faces is moderated by the social character of the task that participants are performing. Thus, the activation of a social processing mode seems to modulate the initial allocation of attention towards angry faces.

## Supplementary Material

nsad070_Supp

## Data Availability

All data and analysis codes as well as the codes of the experimental routines are available at the Open Science Framework. The files can be accessed via the following link: https://osf.io/bx9qe/.

## Footnotes

1 Although it has been shown that the P1 component is amplified for validly cued targets compared to invalidly cued targets, a temporal overlap is likely not problematic in the design of our experiment for two reasons: first, we use non-informative cues in our design. That is, the cue is valid on 50% of the trials and invalid on the remaining 50% of the trials, and this is orthogonalized in regard to the location of the cue. Thus, the target-locked P1 component cannot systematically bias the cue-locked N2pc by design. Second, the increased P1 component for validly cued targets most likely reflects perceptual facilitation. Importantly, in studies where this amplification of the P1 was observed, the target was the only stimulus presented during the target display (Pourtois *et al.*, 2004; Brosch *et al.*, 2008). Therefore, the cue guided attention to a location where no stimulus was presented on invalid trials. In contrast, we use a design where each target is accompanied by a distractor stimulus. Thus, on invalid trials, perceptual facilitation for the distractor might occur.

2 In order to further rule out a possible contamination of the cue-locked N2pc component by the target-locked P1 component (Footnote 1), we additionally analysed the N2pc component in an earlier time window ranging from 150 to 250 ms. This analysis replicated the results of the main analyses. Most importantly, a significant target type × hemisphere interaction occurred, *F*(1, 47) = 4.97, *P* = 0.031, η_p_² = 0.096. Again, mean N2pc amplitudes were larger in the social target condition than in the non-social target condition. Interestingly, the mean N2pc amplitude only differed significantly from zero in the social target condition, *t*(47) = 5.23, *P* < 0.001, *d*_Z_ = 0.75, but not in the non-social target condition, *t*(47) = 1.80, *P* = 0.078, *d*_Z_ = 0.26.

3 One might argue that even in the social target condition, no attentional bias towards angry faces should occur because the equivalent of attentional control settings in the domain of emotional stimuli would be substantially narrower than our definition of a social processing mode: contingent capture in basic attention research argues that attentional control settings are tuned to specific feature values. For example, participants’ attention is only captured by red cues when they are searching for a red target and it is only captured by green cues when they are searching for a green target (Folk and Remington, 1998). If we translate this concept to the domain of emotional stimuli, one expects that participants only show an attentional bias towards angry face cues if they are searching for an angry target face. However, as we showed previously, attentional bias towards angry faces is not contingent on search for an angry target face (Wirth and Wentura, 2018a).
