# Supplementary material for: Social processing modulates the initial allocation of attention towards angry faces: evidence from the N2pc component
Source: Soc Cogn Affect Neurosci. 2023 Nov 16;18(1):nsad070. doi: 10.1093/scan/nsad070 (PMC10689156; doi:10.1093/scan/nsad070)
Supplement: nsad070_Supp [file nsad070_supp.zip › N2pcSocialNonsocial_Supplement_WithFigures.docx]

**Supplementary materials:**

**detailed information on the behavioural pilot study**

Dot-probe studies frequently use a cue-target onset asynchrony (CTOA) of 500 ms (see Bar-Haim et al., 2007, for a review). Despite its prevalence, however, the use of such a long CTOA seems problematic for two reasons given findings from basic attention research on the time course of shifts in covert spatial attention: First, stimulus driven shifts in covert attention peak at approximately 100-150 ms after stimulus onset (Müller & Rabbitt, 1989) and the use of longer CTOAs in the dot-probe task may tap into shifts of overt attention (Cooper & Langton, 2006; Petrova et al., 2013; Stevens et al., 2011; Weierich et al., 2008). Second, when long CTOAs are used in a cueing task, a phenomenon called *inhibition of return* can occur. This phenomenon is characterized by faster RTs on invalid than on valid trial. It is interpreted in terms of the cognitive system inhibiting the scanning of an area that was already attended to in order to avoid redundancies (Lupiáñez et al., 1997; Lupiáñez & Milliken, 1999; Samuel & Kat, 2003).

Therefore, we consistently employed a CTOA of 100 ms in our previous studies (Wirth & Wentura, 2018a, 2019). However, such a short CTOA might be problematic for an ERP study because the N2pc component elicited by the cue stimuli would temporally overlap with the P1 component elicited by the target stimuli. Thus, any modulations of the cue-locked N2pc could be contaminated by modulations of the target-locked P1 (but see Footnote 1 of the main manuscript). Therefore, we decided to increase the CTOA to 200 ms in the ERP study. However, increasing the CTOA makes the detection of RT-based cuing scores potentially less likely. Since the complete absence of RT-based cueing scores would make any interpretation of the results very difficult (see our hypotheses at the end of the introduction of the main manuscript), we decided to run a behavioural pilot study in order to ascertain that significant cueing scores for angry faces (and a significant modulation of these cueing scores by social task demands) also occur with a CTOA of 200 ms.

**Method**

***Participants***

Seventy-eight university students between the ages of 18 to 40 received €20.00 for their participation. Three participants were excluded from all further analyses because their overall accuracy in at least one of the experimental blocks was more than 3 interquartile ranges below the first quartile of the distribution of all participants (Tukey, 1977). Of the remaining *N* = 75 participants, 16 were male and 59 were female. Age ranged from 18 to 40 (*M*= 24.1, *SD* = 4.3). All participants reported normal or corrected-to-normal vision and provided informed consent prior to testing.

The sample size was determined according to the following considerations: based on previous studies (Wirth & Wentura, 2018a, 2019), we expected an effect size of *d*_Z_ = 0.30 for the moderation of the attentional bias towards angry faces by the social character of the targets. According to G*Power (Faul et al., 2007), given an α = .05 (one-sided), 71 participants are needed to detect an effect of this size with a power of 1-ß = .80. We decided to test a few more participants in order to compensate for potential exclusions.

***Design***

We employed a 2 (*target type:* social target vs. non-social target) × 2 (*cue validity:* valid angry cue vs. invalid angry cue) × 2 within-subjects design. Cue validity was randomly varied on a trial-by-trial basis whereas target type was a blocked factor. Furthermore, we measured participants trait anxiety with the trait scale of the German version of the State-Trait Anxiety Inventory (Laux et al., 1981) as a covariate. We did not find any moderation of participants’ attentional bias towards angry face cues in previous studies (Wirth & Wentura, 2018a, 2019). However, since our aim is to investigate the moderation of attentional bias towards angry faces in the general (i.e., predominantly non-anxious) population, we have to ascertain that any potential effects are not solely driven by a few highly anxious participants in our sample.

***Materials***

We employed the same photographs of eight female and eight male individuals showing angry and neutral expressions as in our previous experiments (Wirth & Wentura, 2018a, 2019). We took these faces from the NimStim set of facial expressions (Tottenham et al., 2009) because this database contains each facial expression both with a closed mouth and with an open mouth. For the present study, we only selected angry faces with closed mouths as exposed teeth are a strong perceptual confound of angry expressions that can potentially distort cueing effects in the dot-probe task (Wirth & Wentura, 2018b) Using Adobe Photoshop (Adobe Systems Inc., San Jose, CA), all stimuli were cropped into a standard oval shape concealing hair and external features and were converted to greyscale (see Figure S1). Participants’ trait anxiety was measured with a computerised version of the trait scale of the German version of the State-Trait Anxiety Inventory (STAI; Laux et al., 1981). This self-assessment scale contains 20 items, each scored between 1 (low anxiety) and 4 (high anxiety).

**Procedure**

The study was conducted on five PCs equipped with 17" CRT monitors using a resolution of 1,024 × 768 Pixels, a refresh rate of 100 Hz, and a colour depth of 32 bit. The experimental routine was programmed using Psychtoolbox-3 (Kleiner et al., 2007) for Matlab (Mathworks, Natick, MA).

Participants were seated approximately 65 cm from the monitor and were presented with an instruction screen explaining the experimental procedure. Figure S1 depicts a schematic illustration of a typical trial and the design of the experiment. Throughout the procedure, a grey fixation cross was presented on a black background to maintain participants’ focus on the centre of the screen. To indicate the beginning of a trial, the fixation cross blinked for 100 ms. The fixation cross then remained on screen for a variable interval spanning from 1,000 to 1,400 ms (in steps of 100 ms) to avoid any anticipatory effects. Subsequently, two photographic face cues, one angry and one neutral, were presented laterally for 200 ms. Each face had a size of 4.5 × 6.2 cm (4.0 × 5.5°); the centre-to-centre distance between the faces was 11.1 cm (9.8°). Immediately after the offset of the cues, two white line drawings—one target stimulus and one distractor stimulus—appeared at the cue positions and remained there until a response was given.





**Fig. S1.** Schematic illustration of a typical trial and the design of the pilot study. For the sake of visibility, proportions are not true to scale.

In the social target condition, these stimuli were schematic faces with a neutral expression—the target face had an open mouth (indicated by a double line) and the distractor face had a closed mouth (indicated by a single line). Participants’ task was to indicate the direction in which the nose of the schematic target face pointed (up or down) while ignoring the distractor face. In the non-social target condition, scrambled versions of the schematic faces were presented. These scrambled faces consisted of the same basic features as the schematic faces, but the spatial configuration of those features was altered (i.e., the mouth was located above the nose, one eye and one eyebrow were located below the nose; see Figure S1). Thus, the scrambled schematic faces conveyed the impression of a complex, meaningless pattern surrounded by a circle. Participants’ task was to find the (target) pattern that contained a horizontal double line (corresponding to the open mouth in the social target condition) and indicate whether the arrow in this pattern (corresponding to the nose in the social target condition) was pointing up or down. Moreover, participants were told to ignore the arrow in the (distractor) pattern, which contained only a single horizontal line (corresponding to the closed mouth of the distractor face in the social target condition).

The schematic faces/scrambled faces had a size of 2.8 × 2.8 cm (2.5 × 2.5°) and the centre-to-centre distance between them was 11.1 cm (9.8°). Nose/arrow directions of target and distractor stimuli were uncorrelated, that is, the nose/arrow of the target stimulus pointed in the same direction as the nose/arrow of the distractor stimulus on 50 % of the trials and in the opposite direction on the remaining trials (this was varied orthogonally to the other experimental factors). Participants were asked to respond as fast as possible by pressing the “t” key for “up,” or the “v” key for “down,” on a standard German QWERTZ keyboard. On half the trials, the target stimulus appeared at the location of the angry face cue (valid cue) and on half the trials it appeared at the location of the neutral face cue (invalid cue). Each response was followed by a 500 ms inter-trial interval. If participants made an error or took longer than 1,500 ms to submit a response, they received a 1000 Hz warning tone of 500 ms duration via headphones.

The experiment comprised 448 trials and lasted approximately 35 minutes. Trials were presented in two blocks consisting of 224 trials each—one with schematic faces as target and distractor stimuli and one with scrambled faces as target and distractor stimuli, in a counterbalanced order.^[[1]](#footnote-1)^ Within each block, a self-paced break was included after 112 trials. At the beginning of each block, participants were presented with 32 training trials that were not included in data analysis. At the end of the experiment, participants completed the trait-anxiety scale of the STAI (Laux et al., 1981).

**Results**

Average accuracy rate across all experimental conditions was *M* = 96.7% (SD = 2.5). For the analysis of response time (RT), RTs below 150 ms were excluded, as were RTs more than 1.5 interquartile ranges above the third quartile of the individual participant’s distribution (separately for the two experimental blocks; Tukey, 1977). This led to the exclusion of 1.8% of all trials with correct responses. After outlier removal, average individual RTs for correct responses ranged from *M* = 572 to *M* = 980 ms (grand mean was *M* = 734 ms, *SD* = 87).

We calculated a 2 × 2 within-subjects ANOVA with the factors target type and cue validity and (correct) RTs as the dependent variable. The ANOVA showed significant main effects of target type, *F*(1, 74) = 11.30, *p* = .001, η_p_² = .132, with RTs being shorter in the social target block (*M* = 723 ms, *SD* = 84) than in the non-social target block (*M* = 743 ms, *SD* = 98) and of cue validity, *F*(1, 74) = 7.02, *p* = .010, η_p_² = .087, with RTs being shorter on valid trials (*M* = 731 ms, *SD* = 87) than on invalid trials (*M* = 735 ms, *SD* = 89). Finally, the target type × cue validity interaction was significant, *F*(1, 74) = 3.03, *p* = .043 (one-tailed)^[[2]](#footnote-2)^, η_p_² = .039.

We calculated separate cueing scores for social target trials and for non-social target trials in order to check whether the pattern found in previous studies (significant cueing scores for social target trials but not for non-social target trials) also occurred with a CTOA of 200 ms. Cueing scores were calculated by subtracting average individual RTs of valid trials from average individual RTs of invalid trials. As can be seen in Figure S2, cueing scores for social target trials (*M* = 7 ms, *SE* = 2) were larger than cueing scores for non-social target trials (*M* = 2 ms, *SE* = 2). Cueing scores for social target trials differed significantly from zero, *t*(74) = 3.15, *p* = .002, *d*_Z_ = 0.36, but cueing scores for non-social target trials did not , *t*(74) = 0.68, *p* = .496, *d*_Z_ = 0.08. As in previous studies, adding participants (z-standardised) STAI score as a covariate did not yield any significant effects involving STAI, all *F*s(1, 72) < 3.60, all *p*s > .061, all η_p_² < .048.^[[3]](#footnote-3)^

**Fig. S2.** Average cueing scores for social target and non-social target trials. Cueing scores represent the difference between the average reaction times to invalidly cued trials and validly cued trials (error bars depict the 95% confidence interval).

**Discussion**

In this pilot study, we aimed to replicate the results obtained with a CTOA of 100 ms in previous studies (Wirth & Wentura, 2018a, 2019) with a longer CTOA of 200 ms. As in our previous studies, when participants performed a task that required social processing of the target stimuli, a significant bias towards angry face cues occurred. However, when participants performed a task that did not require social processing, no such bias occurred. Moreover, the cueing scores in the two conditions were significantly different from each other. Thus, we decided to run an ERP experiment with a CTOA of 200 ms.

**References**

Bar-Haim, Y., Lamy, D., Pergamin, L., Bakermans-Kranenburg, M. J., & van IJzendoorn, M. H. (2007). Threat-related attentional bias in anxious and nonanxious individuals: A meta-analytic study. *Psychological Bulletin, 133*(1), 1–24. <https://doi.org/10.1037/0033-2909.133.1.1>

Brosch, T., Sander, D., Pourtois, G., & Scherer, K. R. (2008). Beyond fear: Rapid spatial orienting toward positive emotional stimuli. *Psychological Science, 19*(4), 362–370. <https://doi.org/10.1111/j.1467-9280.2008.02094.x>

Cooper, R., & Langton, S. (2006). Attentional bias to angry faces using the dot-probe task? It depends when you look for it. *Behaviour Research and Therapy, 44*(9), 1321–1329. <https://doi.org/10.1016/j.brat.2005.10.004>

Faul, F., Erdfelder, E., Lang, A.-G., & Buchner, A. (2007). G*Power 3: A flexible statistical power analysis program for the social, behavioral, and biomedical sciences. *Behavior Research Methods, 39*(2), 175–191. <https://doi.org/10.3758/BF03193146>

Kleiner, M., Brainard, D., & Pelli, D. (2007). What's new in Psychtoolbox-3? *Perception, 36*(S), 14.

Laux, L., Glanzmann, P., Schaffner, P., & Spielberger, C. D. (1981). *Das State-Trait-Angstinventar (STAI): Theoretische Grundlagen und Handanweisung*. Beltz.

Lupiáñez, J., Milán, E. G., Tornay, F. J., Madrid, E., & Tudela, P. (1997). Does IOR occur in discrimination tasks? Yes, it does, but later. *Perception & Psychophysics, 59*(8), 1241–1254. <https://doi.org/10.3758/BF03214211>

Lupiáñez, J., & Milliken, B. (1999). Inhibition of return and the attentional set for integrating versus differentiating information. *The Journal of General Psychology, 126*(4), 392–418. <https://doi.org/10.1080/00221309909595373>

Maxwell, S. E., Delaney, H. D., & Kelley, K. (2017). *Designing experiments and analyzing data: A model comparison perspective* (3rd ed.). Routledge.

Müller, H. J., & Rabbitt, P. M. (1989). Reflexive and voluntary orienting of visual attention: Time course of activation and resistance to interruption. *Journal of Experimental Psychology: Human Perception and Performance, 15*(2), 315–330. <https://doi.org/10.1037/0096-1523.15.2.315>

Petrova, K., Wentura, D., & Bermeitinger, C. (2013). What happens during the stimulus onset asynchrony in the dot-probe task? Exploring the role of eye movements in the assessment of attentional biases. *PLOS ONE, 8*(10), e76335. <https://doi.org/10.1371/journal.pone.0076335>

Pourtois, G., Grandjean, D., Sander, D., & Vuilleumier, P. (2004). Electrophysiological correlates of rapid spatial orienting towards fearful faces. *Cerebral Cortex, 14*(6), 619–633. <https://doi.org/10.1093/cercor/bhh023>

Samuel, A. G., & Kat, D. (2003). Inhibition of return: A graphical meta-analysis of its time course and an empirical test of its temporal and spatial properties. *Psychonomic Bulletin & Review, 10*(4), 897–906. <https://doi.org/10.3758/BF03196550>

Stevens, S., Rist, F., & Gerlach, A. L. (2011). Eye movement assessment in individuals with social phobia: Differential usefulness for varying presentation times? *Journal of Behavior Therapy and Experimental Psychiatry, 42*(2), 219–224. <https://doi.org/10.1016/j.jbtep.2010.11.001>

Tottenham, N., Tanaka, J. W., Leon, A. C., McCarry, T., Nurse, M., Hare, T. A., et. al. (2009). The NimStim set of facial expressions: Judgments from untrained research participants. *Psychiatry Research, 168*(3), 242–249. <https://doi.org/10.1016/j.psychres.2008.05.006>

Tukey, J. W. (1977). *Exploratory data analysis*. Addison-Wesley Pub. Co.

Weierich, M. R., Treat, T. A., & Hollingworth, A. (2008). Theories and measurement of visual attentional processing in anxiety. *Cognition & Emotion, 22*(6), 985–1018. <https://doi.org/10.1080/02699930701597601>

Wirth, B. E., & Wentura, D. (2018a). Attentional bias to threat in the general population is contingent on target competition, not on attentional control settings. *Quarterly Journal of Experimental Psychology, 71*(4), 975–988. <https://doi.org/10.1080/17470218.2017.1307864>

Wirth, B. E., & Wentura, D. (2018b). Furious snarling: Teeth-exposure and anxiety-related attentional bias towards angry faces. *PLOS ONE, 13*(11), e0207695. <https://doi.org/10.1371/journal.pone.0207695>

Wirth, B. E., & Wentura, D. (2019). Attentional bias towards angry faces is moderated by the activation of a social processing mode in the general population. *Cognition & Emotion, 33*(7), 1317-1329. <https://doi.org/10.1080/02699931.2018.1561423>

1. Because of the exclusions of participants, 36 participants of the final sample completed the social target block first, whereas 39 participants completed the non-social target block first. [↑](#footnote-ref-1)
2. Since an *F*-test with one df_N_ is equivalent to a *t*-test and because we had specific (i.e., directed) predictions, a one-tailed test is permissible (Maxwell et al., 2017). Note that our power plan (see *Participants*) was based on one-tailed testing. [↑](#footnote-ref-2)
3. Adding STAI as a covariate reduced the degrees of freedom by two (from 74 to 72) because one participant accidentally did not complete the STAI at the end of the session. Thus, this participant had to be excluded from the ANCOVA. [↑](#footnote-ref-3)
